# Anchored protein kinase A signalling in cardiac cellular electrophysiology

**DOI:** 10.1111/jcmm.12365

**Published:** 2014-09-12

**Authors:** Siddarth Soni, Arjen Scholten, Marc A Vos, Toon AB van Veen

**Affiliations:** a Division of Heart & Lungs, Dept of Medical Physiology, University Medical Centre UtrechtUtrecht, The Netherlands; b Biomolecular Mass Spectrometry & Proteomics, Utrecht Institute for Pharmaceutical Sciences and Bijvoet Center for Biomolecular Research, Utrecht UniversityUtrecht, The Netherlands; c Netherlands Proteomics CentreUtrecht, The Netherlands

**Keywords:** PKA, AKAPs, signal transduction, beta adrenergic signalling

## Abstract

The cyclic adenosine monophosphate (cAMP)-dependent protein kinase (PKA) is an elementary molecule involved in both acute and chronic modulation of cardiac function. Substantial research in recent years has highlighted the importance of A-kinase anchoring proteins (AKAP) therein as they act as the backbones of major macromolecular signalling complexes of the β-adrenergic/cAMP/PKA pathway. This review discusses the role of AKAP-associated protein complexes in acute and chronic cardiac modulation by dissecting their role in altering the activity of different ion channels, which underlie cardiac action potential (AP) generation. In addition, we review the involvement of different AKAP complexes in mechanisms of cardiac remodelling and arrhythmias.

## Introduction

The heart is the engine of our circulatory system that by pumping blood throughout the body ensures that oxygen and nutrients are delivered and CO_2_ and waste products are removed as per the requirements of each organ. This pump function is facilitated by a highly orchestrated contraction of all individual cardiomyocytes that form the cardiac muscle of both atria and ventricles. The cardiomyocytes posses a special contractile protein-machinery allowing them to generate mechanical force after being stimulated by an electrical impulse. Every heartbeat depends on this electrical impulse, also known as the action potential (AP), which is spontaneously raised within the sinoatrial node (SAN) that is located in the lateral right atrium 1. The SAN is made up of a small number of specialized cells and is capable of controlling the beating rate of the heart 2. Its activity is modulated through two opposing parts of the autonomic nervous system being the sympathetic system and the parasympathetic system. The parasympathetic system generally tempers heart rate (it decreases the number of APs per period of time) which is mediated through release of the neurotransmitter acetylcholine that increases the threshold for spontaneous AP generation. Opposing this, the sympathetic nervous system increases the rate and force of ventricular and atrial contraction through release of catecholamines likes adrenalin and noradrenaline. These catecholamines stimulate the SAN to increase firing rate of APs.

The sympathetic nervous system comprises of numerous molecular components that make up a large number of important transduction pathways. One of these pathways is the β-adrenergic pathway, which is activated by catecholamines that bind on the extracellular side to various isoforms of the G protein coupled β-adrenergic receptors (β-receptors). Initially two isoforms (β1 and β2) of the β-receptor family were considered important, but with time more isoforms have been detected although it is still questionable whether these isoforms also fulfil functions in the cardiac muscle 3,4. Activation of the β-receptor increases the intracellular concentration of the second messenger cyclic adenosine monophosphate (cAMP) *via* activation of various adenylyl cyclases (AC) by the alpha subunit of the β-receptors. This increase in cAMP triggers activation of cAMP-dependent protein kinase (PKA) and various other cAMP responsive proteins. Activated PKA is able to phosphorylate a large variety of substrate proteins involved in regulation of electrical activity and contractility, such as the KCNQ1 channel 5, voltage-gated L-type Ca^2+^ channels 6, the ryanodine receptor 7, the SERCA inhibitory protein phospholamban 8, myosin binding protein C and P 9 and troponin I and N 10.

Protein kinase A is a ubiquitously present serine/threonine kinase. In its inactive form it is present as a hetero-tetramer composed of two regulatory subunits (PKA-R) and two catalytic subunits (PKA-C). Binding of cAMP to two specific sites on each regulatory subunit causes a conformational change within the tetramer thereby releasing both PKA-Cs thereby rendering them active 11. Based on the pattern of its elution from DEAE-cellulose columns, the PKA enzyme has been classified into two types, PKA type I and type II 12,13. Both subtypes are encoded by in total seven different genes. There are three subunits of PKA-C: Cα, Cβ, Cγ and four of PKA-R; RIα, RIβ, RIIα and RIIβ 14. Given its ubiquitous presence and its numerous targets, the various isoforms of PKA-R and PKA-C are not sufficient to accommodate the complexity and specificity of total cAMP signalling. To regulate activity of the enzyme in a controlled physiological manner, cAMP/PKA signalling within cells is compartmentalized. This is achieved *via* the diverse family of A-Kinase Anchoring proteins (AKAPs).

The AKAPs compose a large family of structurally diverse proteins 15,16, of which thus far 17 subfamilies have been identified in the heart 17. These proteins are responsible for adequate localization of PKA to different compartments of the cardiomyocyte. All AKAPs utilize a small, structurally conserved amphipathic helix of 14–18 residues to interact with PKA-R with nanomolar affinity 18–20. Nuclear magnetic resonance and X-Ray crystallography studies have revealed that the anchoring domain of AKAPs binds to the hydrophobic groove in the docking and dimerization domain of the regulatory subunit of PKA 21,22. Most AKAPs appear to be specific for the RII subunit, but AKAPs with dual specificity, for example D-AKAP2, have also been identified. These dual specific AKAPs bind both to the RI and RII holoenzyme 23,24. Only recently, the first two AKAPs that are entirely RI specific have been identified, SPHKAP and smAKAP 25,26.

**Figure 1:**
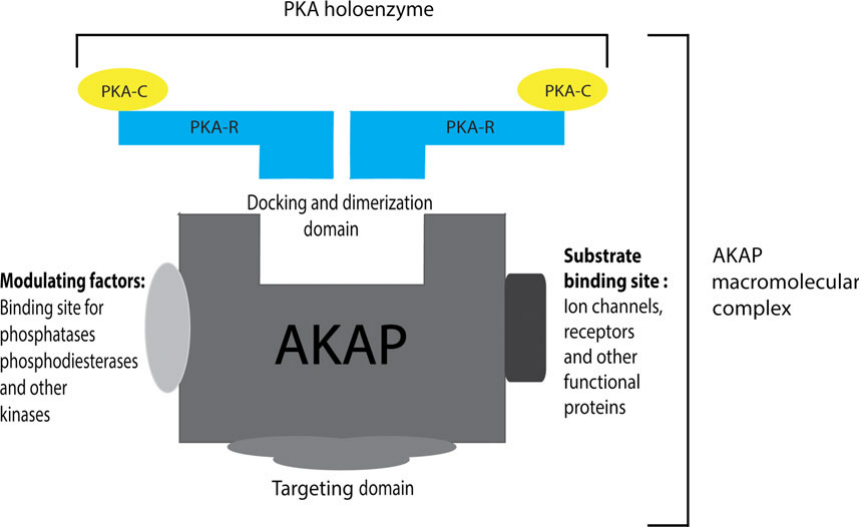
A representation of how an AKAP macromolecular complex is composed. The amphipathic helix of the AKAP binds to docking and dimerization domain of the PKA holoenzyme. The AKAP targets PKA to different substrates *via* the targeting domain and at the same possess sites for bind of other signalling molecules required for the completion of the macromolecular complex.

**Figure 2:**
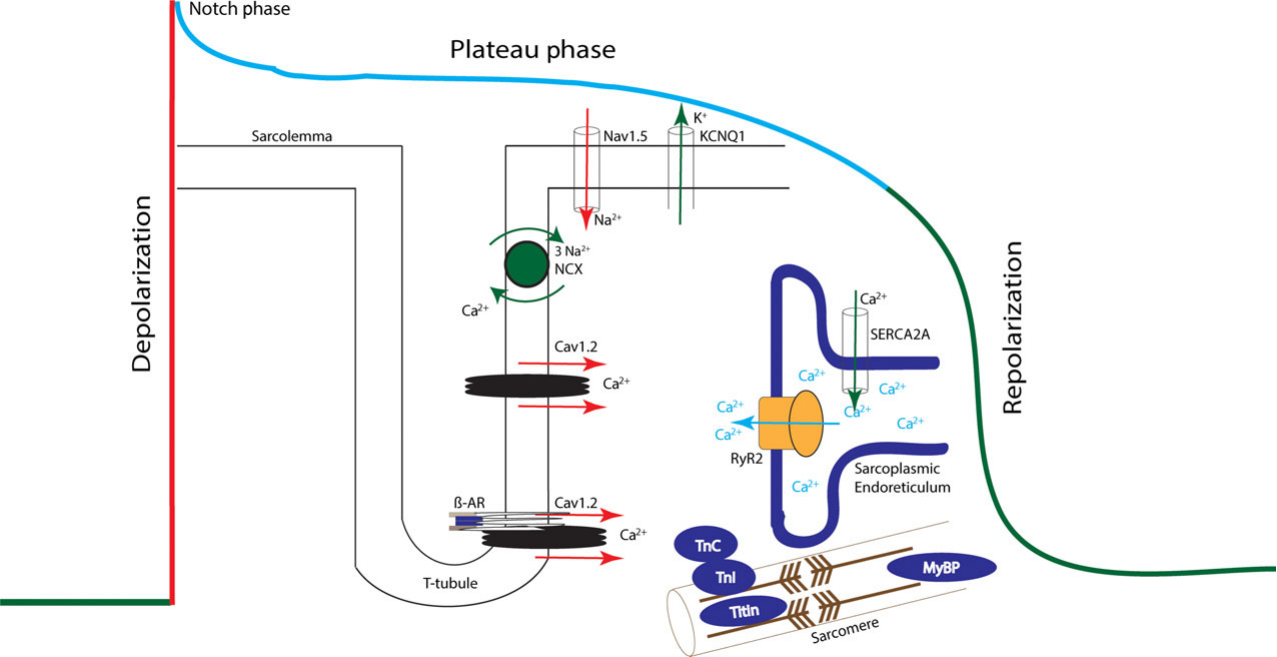
A schematic representation of the four phases of the cardiac action potential. The colour of each phase is correlated with its respective currents by the same coloured arrows. The displayed channels and their currents, and contractile proteins are substrates modulated by PKA phosphorylation during the different phases. The L-type Ca^2+^ channels that are activated in the late-phase of the upstroke are also active during the plateau phase as they deactivate gradually as the resting membrane potential of the cell reaches below their activation threshold. Similarly, the K^+^ channels IK1 and KCNQ1 are also partially open during the plateau phase. For reasons of clarity, this is however not depicted in mingled colours as this figure, in a simplified fashion, illustrates the phase where each of these channels is playing a major role.

As AKAPs are proteins of relatively low abundance, for long it has been quite difficult to study their individual biological functions. However over the past years, a large number of AKAPs have been identified and in addition, their biological functions are currently emerging. In a multi-protein signalling complex, AKAPs assemble PKA together with signal terminators, such as phosphoprotein phosphatases and cAMP-specific phosphodiesterases (Fig. 1). In this review, we will discuss the significance and role of these proteins in controlling the electro-mechanical aspects of normal cardiac function. To do so, we will follow the subsequential sequence of events that underlie AP generation (excitation), an electrical event that triggers contraction of the cardiac muscle. Action potential generation is based on the contribution of several subcellular processes as depicted in Figure 2. In addition, we will briefly touch upon AKAPs that might be involved in maladaptive progression of diverse cardiac pathologies.

## AKAPs and acute cardiac modulation

The first evidence of AKAP-mediated PKA modulation of cardiomyocyte contraction was demonstrated by Fink *et al*. 27. In their study, Ht31, which is a peptide that is based on AKAP-Lbc’s (AKAP13) anchoring domain, was used to disrupt the majority of AKAP–PKA-RII interactions in isolated cardiomyocytes. Ht-31-treated cells showed altered β-adrenergic signalling upon catecholamine stimulation leading to a significantly decreased level of PKA phosphorylation of the sarcomeric proteins Troponin I and myosin binding protein C; proteins which are involved in regulation of actin–myosin interactions. Ht-31 treatment also altered the functional response during β-adrenergic receptor stimulation. The change in cellular function was attributed to the blunted phosphorylation of sarcomeric proteins as only minor changes were observed in intracellular calcium cycling 27. A different but more recent study by Patel *et al*. used a cell penetrating peptide AKAD fused to a carrier peptide TAT to disrupt PKA–AKAP interactions *in vitro* and *ex vivo* thereby significantly inhibiting cellular PKA activity. These disruptions induced a negative effect on chronotropy (rhythm), inotropy (force) and lusitropy (relaxation). On top of that, a decreased PKA phosphorylation of phospholamban was identified, implicating an important role of PKA–AKAP interactions in controlling contractile function throughout the cardiac cycle 28.

Several studies indefinitely show the importance of PKA–AKAP interactions in cardiac contractility. This is illustrated by studies that used introduction of peptides similar to Ht-31, or peptides specific for RI or RII isoforms (such as RIAD 29,30, and AKAPsuper*IS* 31,32, respectively), and small molecules effecting PKA–AKAP interactions 33. From a mechanistic point of view, these studies still had their limitations as they used peptides that knocked out either RII–AKAP or RI/RII–AKAP interactions but not a single specific one. Therefore, these results initiated the scope for studies that aimed to elucidate the roles played by individual AKAP–PKA interactions in cardiac physiology.

### AKAPs and the cardiac action potential

The cardiac AP is characterized by temporal changes in the in-flow and out-flow of different ions *via* their respective ion channels, which in turn elicits the rise and fall of the membrane potential during the four subsequent phases of the AP. All phases of the cardiac AP are influenced through several underlying molecular components like ion channels, modulating kinases and phosphatases, and phosphodiesterases (PDE). One of the ways these molecular components are modified is through phosphorylation. PKA is one of the major kinases phosphorylating several of the proteins that constitute the ion channels when the cardiac AP is acutely affected during catecholamine stimulation. Figure 2 displays a schematic representation of the different phases of the AP, the various underlying currents and molecular components modified by PKA phosphorylation during these different phases. Table 1 lists the different ion channels and their respective site of phosphorylation by PKA.

**Table 1:** List of various ion channels involved in the cardiac action potential, which, are PKA substrates. Phospholamban, though not belonging to the class of ion channels has been included in this classification because of its vital role in the regulation of SERCA

Current	Gene for α subunit	Site of phosphorylation	References
I_na_	SCN5A	Ser525, Ser528 (Human)	34,35,82
I_cal_	Cav1.2	Ser1928	83
SR Ca^2+^ release	Ryanodine Receptor	Ser2030, Ser2808	44,45
SERCA uptake	Phospholamban	Ser16	84
I_Ks_	KCNQ1	Ser27	85
I_kr_	HERG (KCNH2)	Ser283, Ser890, Ser1137, Thr895	86–88
I_Kur_	Kv1.5 (KCNA5)	Ser24	89,90
I_K1_	Kir2.1 (KCNJ2)	Ser425	91
	Kir2.2 (KCNJ12)		
I_K-ATP_	Kir6.2 (Bir JCNJ11)	Thr224	92
I_*f*_	HCN4	Ser719, Ser831, Ser918, Ser1005, Ser1051, Thr1071, Ser1128, Thr1153, Ser1154 and Ser1155	93

#### Role of AKAPs during depolarization

Cardiac contraction is initiated by a fast depolarization of the membrane caused by opening of the Nav1.5 (sodium) channels, subsequently followed by opening of voltage-dependent L-type Ca^2+^ channels. In rats, the cardiac Nav1.5 channel that conducts the depolarizing sodium current (*I*_na_) is phosphorylated by PKA at Ser526 (525 in human) and Ser529 (528 in human) during stimulation with catecholamines 34,35. Logically, the fact that Nav1.5 is phosphorylated by PKA allows one to speculate about the presence of an AKAP that may be mediating the localization of PKA near the sodium channel.

Till date no AKAP has been discovered that is involved in PKA regulation of the cardiac sodium channel. However, a few studies have demonstrated that brain sodium channels (familiar to the cardiac one) are phosphorylated by PKA 36, and one study identified AKAP15 (not specifying which isoform) to be associated with brain sodium channels to tether PKA in close proximity of the target phosphorylation site S673 37. Cantrell *et al*. used an AKAP15 derived peptide, AP2, that disrupted the interaction between AKAP15 and PKA-RII and this disruption resulted in a 15–20% decrease in peak sodium current implying the importance of AKAP15 in PKA regulation of brain-type sodium channels 38. These results were not completely conclusive as the AP2 peptide is expected to bind to PKA-RII and hence to disrupt interactions between more than just one AKAP known to interact with PKA-RII.

During the second half of the depolarization phase the initiation of excitation–contraction coupling (EC) starts through the entry of Ca^2+^ into the cytosol *via* opening of L-type calcium channels that conduct *I*_Ca-L_. During exposure to exercise or stress (also named *the fight-or-flight response*), it is required that the heart beats in a faster rhythm (increased chronotropy) and with a higher force of contraction (increased inotropy) to meet the increase in circulatory demands. To facilitate this, more calcium must be introduced in the cell and/or the Ca^2+^ sensitivity of the ryanodine receptor (Ryr) has to be increased to increase the release of Ca^2+^ from the SR. This requirement is fulfilled by activation of the β-adrenergic receptors and their translating downstream pathways.

**Figure 3:**
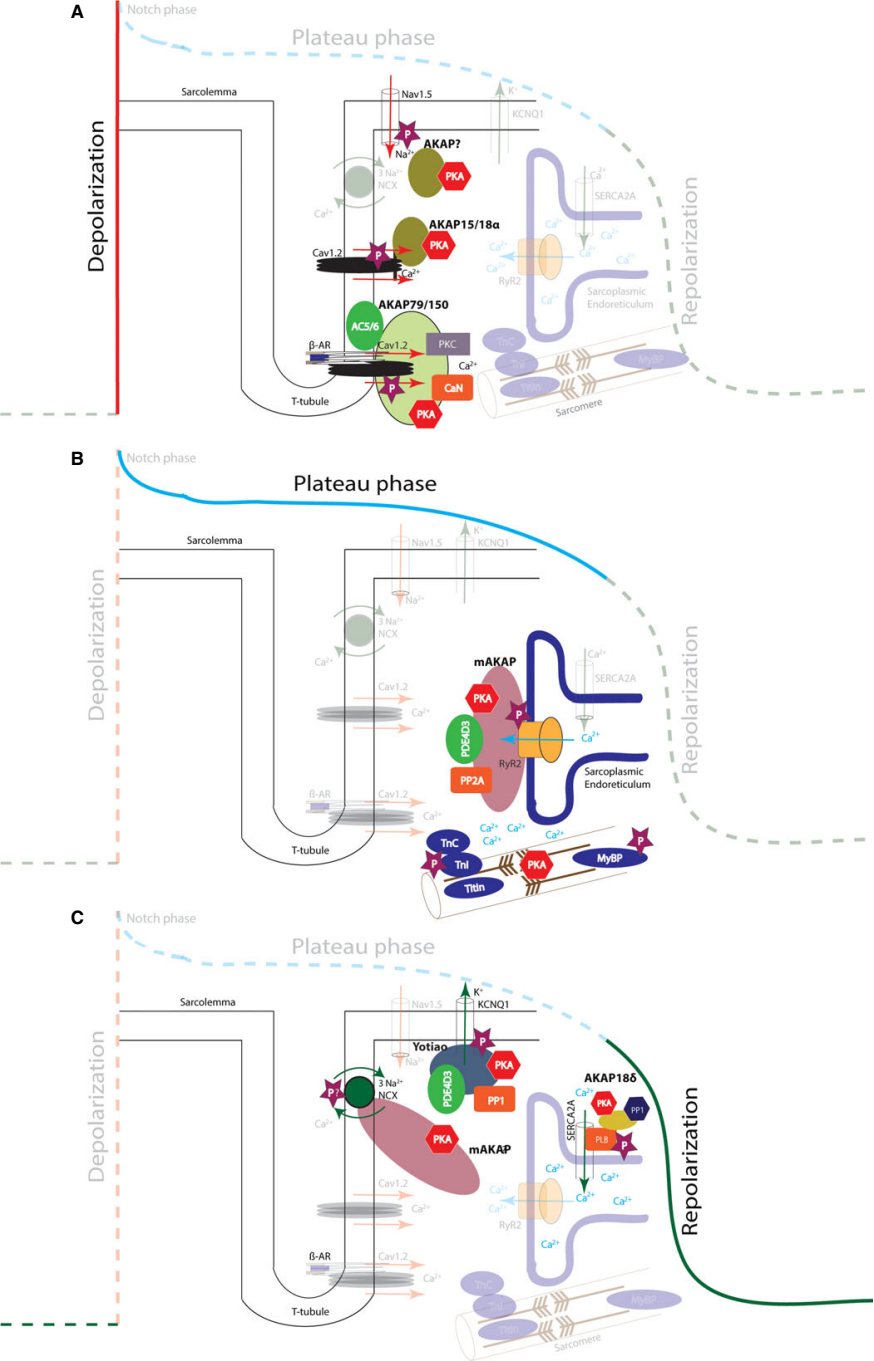
(**A**) Schematic representation of the AKAP-associated protein complexes in the depolarization phase of the AP. AKAP15/18α and AKAP79/150 localize macromolecular complexes to different pools of L-type Ca^2+^ channels. (**B**) mAKAP maintains a signalling complex at the RyR to increase the receptors’ sensitivity to calcium-induced calcium release during sympathetic stimulation. (**C**) Different AKAP-associated complexes function during the repolarization phase of the AP.

AKAP15 (AKAP18α) has been shown to co-immunoprecipitate and co-localize with PKA and L-type Ca^2+^ channels (or C_av_1.2 channels) in rat hearts 39. This interaction of the channel and AKAP15 is mediated through a leucine zipper motif and assists PKA in phosphorylation of the Cav1.2 channel at Ser1928 (Fig. 3A). Inhibition of this complex using competing peptides to disrupt PKA anchoring to C_av_1.2 attenuated the response of the channel to β-adrenergic stimulation by effectively inhibiting the PKA regulation of I_Ca-L_ 39. Following this observation, not unexpectedly, a role of this complex was confirmed in the molecular mechanism that regulates calcium channel function during the *fight-or-flight response* 40, again underlining the importance of the AKAP in excitation–contraction coupling during sympathetic stimulation.

AKAP5 (murineAKAP150, humanAKAP79) has also been identified to direct PKA-dependent phosphorylation of L-type Ca^2+^ channels resulting in augmentation of the current in HEK293 cells transfected with the channel 41. Recently, more detailed information on the exact nature of this regulation in the heart was reported 42. In mice, a macromolecular complex consisting of β-adrenergic receptors, AC5, AC6, calcineurin, Caveolin-3, PKA, AKAP5 and a subset of L-type Ca^2+^ channels was identified (Fig. 3A). The composition of this complex was disrupted in cardiomyocytes isolated from AKAP5 knockout mice and consequently, β-adrenergic regulation of Ca^2+^ transients and phosphorylation of PKA substrates involved in Ca^2+^ cycling was disturbed 42.

#### Role of AKAPs in the plateau phase

As the AP is reaching the plateau phase, EC coupling enters its second phase in which Ca^2+^ is progressively released from the SR *via* ryanodine receptors through a process named the *calcium-induced calcium-release* mechanism. Upon sympathetic stimulation, phosphorylation of the ryanodine receptor increases its sensitivity to the increased *I*_Ca-L_ resulting in an enhanced SR Ca^2+^ release. This allows a stronger force of contraction by making more Ca^2+^ available to bind to the myosin and troponin filaments 43. The ryanodine receptor is multi-phosphorylated by different kinases, for example at Ser2030, Ser2808 and 2809 by PKA. Phosphorylation by PKA at Ser2030 and 2808 has identified these serine residues as the functionally important ones, and also the ones playing a role in disease mechanisms 44,45. Phosphorylation of the receptor during adrenergic stimulation is performed by PKA that is anchored to the receptor by mAKAP (muscle specific AKAP), a member of the AKAP6 gene family 46,47. mAKAP also maintains the balance in PKA activity and ryanodine phosphorylation by additionally anchoring PDE4D3, a PDE that keeps a check on the local cAMP levels to preserve a balanced activity of the enzyme. Moreover, PP2A is also anchored which dampens the phosphorylation status of the ryanodine receptor 7,48 (Fig. 3B). A recently conducted study stressed the involvement of AKAP5 playing a role in phosphorylation of the ryanodine receptor although the underlying mechanism still remains unclear 42.

#### Role of AKAPs during repolarization

The plateau phase is followed by the repolarization phase and this phase is executed through two different movements of ions: (*i*) removal of Ca^2+^ from the cytosol and (*ii*) extrusion of K^+^ from the cell to restore the cells’ negative membrane potential.

*Role of AKAPs during removal of cytosolic Ca*^*2+*^. During the final part of EC coupling, cytosolic Ca^2+^ levels decline to arrest contraction and to initiate relaxation of the cardiomyocytes. The removal of cytosolic Ca^2+^ is achieved *via* four mechanisms; through the sarcoplasmic reticulum Ca^2+^ ATPase (SERCA), the sarcolemmal Na^+^/Ca^2+^ exchanger (NCX), the sarcolemmal Ca^2+^ ATPase and the mitochondrial Ca^2+^ uniporter (reviewed by Bers *et al*. 49). SERCA regulates Ca^2+^ re-uptake into the SR and in its inactive form it is bound to a small 52 amino acid inhibitory protein named phospholamban. Phospholamban is bound to SERCA in a dephosphorylated state and its inhibitory function can be modulated *via* phosphorylation by PKA. Once activated, phospholamban dissociates from SERCA thereby increasing the activity of SERCA 50. A complex composed of AKAP18δ, PKA, phospholamban and SERCA has been identified and it has been shown that AKAP18δ plays a vital role in phosphorylation of phospholamban upon adrenergic stimulation 8. In that study, disruption of the interaction between phospholamban and AKAP18δ reduced PKA-dependent phosphorylation of phospholamban by 50%. Moreover, neonatal rat ventricular myocytes with a siRNA mediated complete knockdown of AKAP18δ, showed decreased rates of Ca^2+^ re-uptake by SERCA during β-adrenergic stimulation *via* isoproterenol. These results taken together highlight the importance of this AKAP in the final phase of EC coupling of the cardiomyocyte (Fig. 3C). However, a recent study by Jones and colleagues questions this model 51. In their study, the authors show a normal regulation of calcium cycling in cardiomyocytes, and an unaffected phosphorylation of phospholamban at the PKA site Ser16 in an AKAP7 (all isoforms of the family, AKAP18α, β, γ and δ) knockout mouse model. As such they claim the involvement of a different, but yet unknown, AKAP. Results of this latter study raise questions, which require re-establishment of the previously accepted model.

The NCX also plays a vital role in the efflux of Ca^2+^ from the cells after contraction. It extrudes one Ca^2+^ in exchange for three Na^+^. Schulze *et al*. showed the existence of a macromolecular complex in the adult rat heart, which was composed of mAKAP, PKA-RI, PP1, PP2A, PKC and the NCX channel (Fig. 3C) 52. In addition, Western blot experiments on NCX immunoprecipitations excluded the interaction in that complex of five other tested AKAPs (AKAP79, AKAP95, AKAP149, AKAP121 and AKAP220). It is widely accepted that mAKAP is a RII specific AKAP as it lacks the docking domain to bind PKA-RI. Surface plasmon resonance studies that are able to uncover direct interactions between proteins confirmed this as no interaction between mAKAP and the RI subunit could be revealed 53. Surprisingly, in the study by Schulze *et al.,* the authors could not confirm the presence of the RII subunit in their macromolecular complex although the unexpected RI subunit was found 52. As these findings were based on co-immunoprecipitation and co-localization, it is still possible that the presence of both proteins in the complex is based on indirect interactions. As such, the role of PKA in phosphorylation of NCX still remains unspecified.

Though not much is known about the modulation of the mitochondrial uniporter through phosphorylation by PKA, the sarcolemmal ATPase is phosphorylated by PKA and this phenomenon is important for the increased outward movement of intracellular Ca^2+^ 54. Untill date, no AKAP has been discovered that might be involved in such a possible macromolecular complex around the sarcolemmal Ca^2+^ ATPase.

*Extrusion of K*^*+*^. After the removal of Ca^2+^ from the cytosol through SERCA and the other named mechanisms has been completed, relaxation starts and the AP proceeds towards the final repolarization phase. The L-type Ca^2+^ channels start to close whereas the delayed rectifier conducting channels, which are still open, ensure a progressive hyperpolarization of the membrane potential. This leads to opening of more K^+^ channels like Kir2.1 channels (conducting *I*_K1_). The outward flow of K^+^ ions causes the cell to repolarize and most channels close when the membrane potential reaches about −85 mV. The repolarization phase ensures ventricular relaxation to facilitate an appropriate diastolic filling of the cardiac chambers. The KCNQ1 channel, which conducts the repolarizing *I*_Ks_ current is phosphorylated by PKA at Ser27 and this phosphorylation is modulated by a channel micro domain formulated by a macromolecular complex of PKA, KCNQ1, PP1, PDE4D3 and the anchoring protein yotiao (AKAP9) 5. Yotiao mediates anchoring of PKA to the KCNQ1 channels to modulate channel function upon β-adrenergic stimulation when a higher rate of contraction and a faster relaxation is required (Fig. 3C). In this complex, PDE4D3 and PP1 are present to maintain equilibrium in PKA activity and to monitor the phosphorylation of the KCNQ1 channel respectively 55.

Yotiao itself has been implicated in post-phosphorylation and allosteric modulation of the KCNQ1 channel. Phosphorylation of yotiao by PKA at Ser43 has been shown to be critical for KNCQ1 channel function 56. Replacement of Ser43 by an alanine not only disrupted the PKA-dependent phosphorylation of yotiao at the N-terminus but also diminished the functional response of the KCNQ1 channel (wild-type and pseudo-phosphorylated) to cAMP, stressing the functional importance of this AKAP.

Apart from Yotiao, there are two other AKAPs that have been shown to be phosphorylated by their associated kinases. Gravin (also known as AKAP 250) binds PKA and PKC and is a substrate for these kinases 57,58. Phosphorylation of Gravin seems to affect the effectivity of the complex to modulate β2-adrenal receptor function 58. Secondly, AKAP-Lbc, which synchronizes the activation of PKD, is another AKAP phosphorylated by PKA 59,60. Phosphorylation of AKAP-Lbc on serine 1565 recruits the protein 14-3-3 to the complex which, upon binding, is able to inhibit the Rho-guanine nucleotide exchange factor (GEF) activity of AKAP-Lbc 59. In general, the functional effects of phosphorylation of AKAPs by its associated kinases is largely unresolved, but these studies do imply that phosphorylation of AKAPs potentially can modulate association with their respective substrates or affect subcellular localization.

## AKAPs and chronic cardiac remodelling

The PKA signalling pathway and especially alterations in this signalling pathway are highly associated with cardiac remodelling during situations that deviate from normal physiology (*e.g*. cardiac hypertrophy and failure). Cardiac hypertrophy is either a physiological response of the heart (compensated) to chronically increased systemic demands, but may also manifest itself as a pathophysiological (decompensated) form. Recent studies (see Table 2) have revealed (*i*) the involvement of two AKAPs, AKAP-Lbc and mAKAP 61–68 and (*ii*) altered organization of PKA–AKAP complexes in the hypertrophic response of the heart 71,72. In addition, AKAPs have also been involved in electrical remodelling and arrhythmogenesis 5,76,79–81.

**Table 2:** Summary of AKAPs involved in different cardiac diseases

Cardiac disorder	AKAP isoform	References
Cardiac rhythm disorder	D-AKAP2 (AKAP10)	80,81
Long-QT syndrome	Yotiao (AKAP9)	76
	AKAP150 (AKAP5)	79
Cardiac hypertrophy	AKAP-Lbc (AKAP13)	61–68
	mAKAP (AKAP6)	
Heart failure	AKAP1, PALM-AKAP2, AKAP7, SPHKAP, MAP2, Yotiao, AKAP13	71,72

### AKAP-Lbc

AKAP-Lbc, a member of the AKAP13 gene family has been identified as a mediator of cardiac hypertrophy 61. Analysis of tissue samples from patients with hypertrophic cardiomyopathy revealed a twofold increased level of AKAP-Lbc in comparison to matched controls. AKAP-Lbc recruits a complex consisting of PKA, PKD1 and PKC. By synchronizing phosphorylation of PKC and PKA (serine 2737), PKD1 is activated and released from the complex to phosphorylate the class II histone deacetylase 5 (HDAC5). In turn, phosphorylated HDAC5 promotes the transcriptional activation of genes involved in hypertrophy *via* the myocyte-specific enhancer-binding factor-2 (MEF-2) pathway 62. This mechanism of AKAP-Lbc/PKD1/HDAC5-mediated hypertrophy has been shown to be involved in compensated hypertrophy. Mouse models of pathological hypertrophy with a truncated version of the AKAP-Lbc that disrupts its interaction with PKD1 exhibited an accelerated progression to cardiac dysfunction with attenuated compensated hypertrophy and reduced levels of HDAC5 phosphorylation. This study further confirms the role of AKAP-Lbc in cardiac remodelling 63.

AKAP-Lbc, in addition, acts as a GEF for the small GTPase RhoA that is involved in pathways leading to cardiomyocyte hypertrophy. Appert-Collin *et al*., showed an increase in AKAP-Lbc mRNA levels and heart weight/bodyweight ratios after chronic infusion of phenylephrine into mice 64. Knock-down of AKAP-Lbc *via* RNA interference in isolated cardiomyocytes attenuated this hypertrophic response. To elucidate the downstream effectors of this pathway, a recent study by Vescovo *et al*. demonstrated a transduction complex formed by AKAP-Lbc and the inhibitor of NF-κB kinase subunit β (IΚΚβ) 65. They showed that AKAP-Lbc promotes the formation of RhoA-GTP and activation of Rho kinase, which leads to activation of NF-κB. Activated NF-κB, in turn, induces transcription of the interleukin-6 gene (IL-6) ensuing the stimulation of IL-6-mediated pathways involved with foetal gene expression and cardiac hypertrophy.

### mAKAP

Elevated mAKAP expression levels have been detected in cultures of neonatal rat cardiomyocytes treated chronically with hypertrophic stimulants like phenylephrine 66. This anchoring protein indirectly mediates the activation of the Ca^2+^/Calmodulin-dependent phosphatase calcineurin. Calcineurin’s activity is enhanced through elevation of cytoplasmic Ca^2+^ levels resulting from PKA-dependent phosphorylation of the ryanodine receptor. Activated calcineurin subsequently dephosphorylates the hyper-phosphorylated nuclear transcription factor NFATc, an event that leads to its translocation to the nucleus where it mediates the expression of hypertrophic genes. During pathological remodelling, AC5 associates with mAKAP and this complex proved to be important in cardiomyocyte hypertrophy by controlling the regulation of the second messenger cAMP 66,67. A recent study indicates that mAKAP also binds to phospholipase-c (PLC) and disruption of this complex in neonatal cardiomyocytes inhibits endothelin-1-induced hypertrophy 68.

### Altered organization of AKAPs

During heart failure and other cardiomyopathies, several neurohumoral factors are deregulated in the heart. Disoriented catecholamine signalling leads to an imbalance in the β-adrenergic pathway by desensitization and down-regulation of β-adrenergic receptors and hence disturbed cAMP/PKA signalling 69,70.

A recent chemical proteomics-based study that we performed showed a completely reorganized profile of PKA–AKAP interactions in patients with end-stage heart failure in comparison to healthy individuals 71. Though the mechanisms behind this reorganization and its implications for, and contribution to cardiac dysfunction are still not fully understood, it strengthens the suggestion that disturbed PKA–AKAP-mediated β-adrenergic signalling is involved in the failing heart.

Earlier studies have shown that RII auto-phosphorylation enhances its binding to Ht31 (which represents the RII-binding domain of AKAPs). Showing a decreased auto phosphorylated RII in the failing human heart allows to hypothesize that under these conditions a decrease in auto-phosphorylation of RII may hamper targeting of RII (because of decreased RII–AKAP interactions) and subsequently to a decreased/ineffective phosphorylation levels of PKA substrates 72.

To further study these abnormalities in PKA–AKAP signalling pathways, we recently conducted a time-based study exploring the PKA–AKAP interaction profile during the different phases (compensated, transition and early heart failure) in progression to heart failure 73. In a rat model of pressure overload, the compensated phase showed an increased association of AKAPs with the R subunits. This was accompanied by a subsequent increase in PKA substrate phosphorylation and PDE2A association, the latter as a possible element to keep PKA activity levels under control. However, in the transition phase, AKAP association was decreased and so was the level of PKA substrate phosphorylation. In contrast, R1 and RII subunit association was increased suggesting an attempt of the system to compensate for the decreased PKA substrate phosphorylation that was caused by a decreased targeting of R subunits. Finally, the stage of early heart failure showed a further decrease in AKAP association levels, PKA substrate phosphorylation and decreased R subunit levels compared to the transition stage suggesting an exhaustion of the PKA–AKAP signalling system. Decreased PKA substrate phosphorylation has been witnessed in human heart failure and the above described results do match with those of Aye *et al*. and Zakhary *et al*. 71,72,74.

### AKAPs and long-QT syndrome

Long-QT syndrome (LQTS) is an inherited arrhythmogenic disease characterized by prolongation of the QT interval on the echocardiogram (ECG). The QT segment of the ECG relates to the repolarization phase of the AP, which points out the rate of ventricular relaxation during diastole. Patients suffering from LQTS are vulnerable to sudden cardiac death during exercise or stress because of repolarization lability 75. As discussed earlier, a macromolecular complex of PKA, KCNQ1, Yotiao, PP1 and PDE4d3 is necessary for the enhanced functioning of this *I*_ks_ encoding KCNQ1 channel to increase current amplitude during sympathetic regulation 5. Single nucleotide polymorphisms (SNP) in both the KCNQ1 and the Yotiao genes are described that lead to a dissociation of the Yotiao-macromolecular complex from the KCNQ1 channel and this is known to cause LQTS1 76.

The molecular nature of the binding between Yotiao and KCNQ1 involves the distal C-terminus of the channel and both the C and N termini of Yotiao. A rare missense mutation in a LQTS family was discovered in yotiao (S1570L) 76. Analysis of the consequences of this mutation in those patients using a computational model of a ventricular myocyte predicted a disrupted binding of the anchoring protein to the channel. The result of this disruption is a markedly reduced PKA phosphorylation of the channel and a blunted response of the channel to cAMP. This leads to an insufficient AP/QT shortening and an increased QTc (QT interval corrected for heart rate) during adrenergic stimulation as was observed in this particular family 76. A similar molecular phenotype was observed with the G589D missense mutation found in the docking domain of KCNQ1 77.

A different form of LQTS is LQTS8 (Timothy syndrome), which is characterized by a single amino acid substitution (G406R) in the Cav1.2 L-type Ca^2+^ channel. This results in an abnormally slow rate of inactivation and exhibition of a high frequency of coordinated openings between nearby channels 78. Cheng and colleagues recently showed that AKAP150 (AKAP5 family), that steers regulation of Cav1.2 during EC coupling 42, anchors a complex composed of PKA, AC5 and calcineurin 79. AKAP150 is required for the presentation of a LQT8 phenotype in a transgenic mouse model of this disease. The mutation of these LQT8 Cav1.2 channels resulted in cardiac hypertrophy, delayed inactivation of I_*ca-L,*_ increased coupled gating of the Cav1.2 channels and increased AP durations. This phenotype could be rescued by crossing the LQT8 line with AKAP150^−/−^ knockout mice, which resulted in a phenotype almost comparable to that in wild-type control mice 79.

### AKAPs and aberrancies of cardiac rhythm

Two separate studies suggest that the dual specific mitochondrial D-AKAP2 (AKAP10) is involved in regulation of heart rhythm 80,81. The exact molecular mechanisms behind this still remains to be elucidated, but findings conducted in humans, mice and mouse embryonic stem cell derived embryoid bodies have a similar inference 80. The results of a screen, comparing allele frequencies of 6500 SNP between DNA pools of healthy European-American individuals showed a SNP that results in an amino acid change at 646 from Isoleucine (Ile) to Valine (Val) in D-AKAP2 81. This amino acid SNP was located within the PKA-binding domain of the protein and *in vitro* assays demonstrated that the Val variant bound PKA-RIα with a threefold lower affinity than the Ile variant. In their study, the investigators also revealed that the Val variant was associated with a significant shortening of the P-R interval on the ECG. Although the experimental evidence was not provided, the authors speculated that the effect on electrical characteristics of the Val variant could be attributed to enhanced localization of PKA-RIα to the plasma membrane. Through its PDZ binding motif in the C-terminus, D-AKAP2 tentatively would be able to anchor to ion channels and by the increased affinity of PKA-RIα, this could result in an enhanced phosphorylation of ion channels thereby altering the electrical characteristics in individuals expressing the Val variant. Examination of the Val646 polymorphism in a different cohort of 122 patients showed that these patients with the Val646 SNP had elevated heart rates, but low heart rate variability, which is a known risk factor of triggering sudden cardiac death 80.

## Conclusion and futures perspective

The importance of AKAPs for PKA efficiency and specificity in space and time is evident. However, the functional characterization of all individual cardiac AKAPs is far from complete. This lack of knowledge is of functional importance for the field of cardiac electrophysiology. Several ion channels that play a major role in the morphology of the cardiac AP are functionally modulated PKA substrates (as shown in Table 1), which implies the involvement of AKAPs and the current absence of knowledge of these plausible AKAPs opens new avenues to explore. This information could further strengthen our understanding of ion channel dysfunction in pathophysiology in situations where expression levels seem to be unchanged, but current densities appear to be affected. Inappropriate phosphorylation status potentially in combination with disturbances in protein turn over/mislocalization may result from defective composition of PKA/AKAP complexes as we have shown before in the setting of human end-stage heart failure 71. Moreover, it appears that in the subsequent phases that precede end-stage heart failure composition and functionality of the macromolecular complexes that embed the different AKAP isoforms dynamically changes 73. During these different phases, also the current densities of PKA-sensitive ion channels are affected which contributes to differential sensitivity to pro-arrhythmia.

Further knowledge on the AKAP family of proteins in relation to modulation of electrophysiological characteristics might be acquired through lessons that can be learnt from their role as established in signalling mechanisms of neuronal ion channels. Discovery of these proteins and their functions will help to understand the complex matrix of signalling mechanisms in the heart and this will subsequently help us to understand cardiac diseases in more detail at the molecular level. β-blockers have been effective drugs to manage heart failure, but upon chronic administration act by blunting the total β-adrenergic/cAMP response. Preferably, treatment would be optimized if target specific interventions would be possible within the plethora of events that are regulated *via* with the adrenergic signalling. Although still far away from clinical applicability, in-depth knowledge of the different functionalities of individual AKAP pathways might ultimately contribute to development of more specific drugs to achieve clinically more effective treatments with less side effects. At first glance, a peptide like Ht-31 (mimicking the docking domain which is highly conserved) seems not able to contribute to future drug development as it generally blocks the interaction between several AKAPs and PKA-RII and as such does not provide specificity. Modifications of this peptide, however, might dose-dependently fine tune the blocking capacity and as such differentiate in complex-formation of different AKAP with PKA. To speculate, generation of peptides that mimic the interaction domain of a specific target protein (*e.g*. SERCA or the L-type calcium channel) that binds to one of the AKAP isoforms would also provide a strategy to specify interference. As mentioned, currently this is food for thought and future research has to uncover the potential usefulness.
